# Diagnosis and management of bilateral and multiple semicircular canal dehiscence: a case report

**DOI:** 10.11604/pamj.2025.51.12.47421

**Published:** 2025-05-14

**Authors:** Taha Benatiya Andaloussi, Mohamed Bouqes, Naouar Ouattassi, Mohamed Afellah, Mohammed Ridal, Najib Benmansour, Zouheir Zaki, Abdellatif Oudidi, Mohamed Noureddine El Amine El Alami

**Affiliations:** 1 Otorhinolaryngology Head and Neck Surgery Department, Medicine and Pharmacy Faculty, Hassan II University Hospital, Sidi Mohamed Ben Abdellah University, Fez, Morocco

**Keywords:** Superior and posterior semicircular canal dehiscence, Tullio phenomenon, videonystagmography, temporal bone CT scan, case report

## Abstract

Semicircular canal dehiscence (SCCD) is a rare inner ear disease caused by an anatomical defect in the bony covering of the semicircular canal (SCC). This condition most commonly affects the superior semicircular canal, and less frequently involves multiple canals in one or both ears. Although the clinical and physiological features of a single SCCD are well known, there are only a few reported cases of multiple semicircular canal dehiscences. Thus, their clinical and physiological characteristics require further investigation. We present the case of a 59-year-old male patient of Moroccan ethnicity who presented with chronic imbalance and mild dizziness, induced by loud noises. As a dentist, he experienced extreme difficulty while drilling his patients' teeth, exhibiting nausea, tachycardia, and sweating. Clinical oto-vestibular and neurological examinations, tonal audiometry, and videonystagmography (VNG) revealed peripheral vestibular syndrome. Bilateral multiple SCCD was subsequently confirmed using high-resolution computed tomography imaging of the temporal bone. The clinical manifestations in this case were primarily neurovegetative symptoms of vestibular dysfunction. Functional assessments revealed vibratory-induced down-beating nystagmus and left preponderance on caloric testing. Computed tomography (CT) imaging confirmed dehiscence in the posterior and superior semicircular canals on the right side, and posterior semicircular canal dehiscence on the left side.

## Introduction

Semicircular Canal Dehiscence Syndrome (SCDS) is a rare pathology of the inner ear characterized by an anatomical defect of the bone covering the semicircular canals. First described by Minor *et al*. in 1998 [1], it can lead to various symptoms such as positional vertigo, increased susceptibility to auditory stimuli, tinnitus, and balance disorders. This condition primarily affects the Superior Semicircular Canal (SSC), and less frequently the Posterior Semicircular Canal (PSC) in one or both ears. Although the diagnosis of SCDS can be complex due to its variable presentation, videonystagmography (VNG) has become an essential tool to help establish an accurate diagnosis. The clinical manifestations and VNG features of a single SSC are well known. However, there are only a few cases of multiple SCC dehiscence, and therefore, their clinical and functional characteristics require further investigation. This article examines VNG results in a case of bilateral and multiple SCCD.

## Patient and observation

**Patient information:** we present the case of a 59-year-old Moroccan male who presented to the outpatient clinic with recurrent vertigo and increased sensitivity to sounds, especially since he is a dentist, and he is exposed to loud noise while drilling his patients´ teeth. The patient reported a progressive bilateral hearing loss. He also experienced an imbalance since 2008, associated with recurrent vertigo worsened by head movements. Furthermore, he had many imbalance episodes triggered by loud sounds (Tullio phenomenon), associated with nausea, tachycardia, and sweating. We performed a comprehensive evaluation, including clinical examination, tonal audiometry, VNG, and radiology investigations to set up the diagnosis.

**Clinical findings:** otoscopy was uneventful, complete vestibular examination disclosed only an abnormal Unterberger´s stepping test, which showed rotation to the right. Neurological examination was normal. In fact, we found no motor, sensory, or coordination impairment.

**Figure 1:**
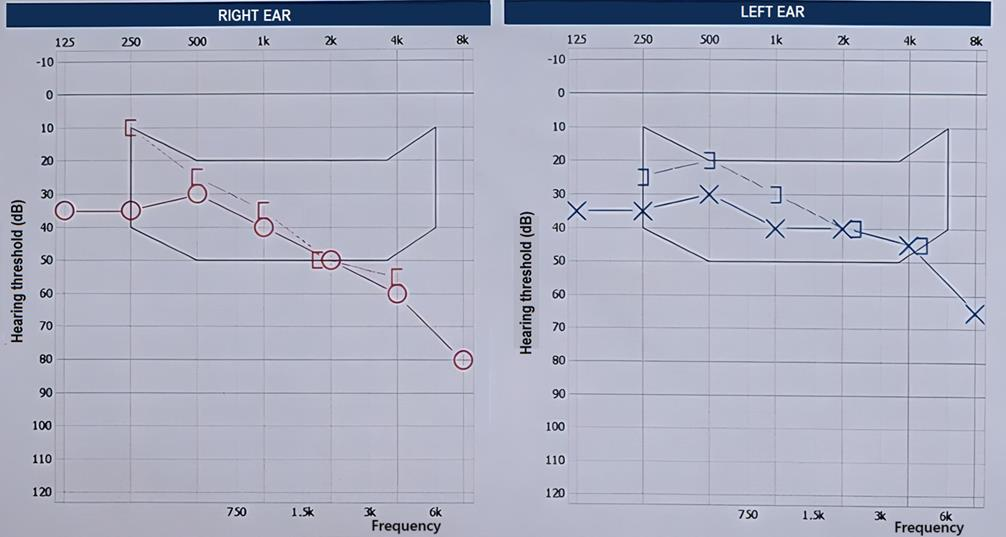
tonal audiometry reveals a bilateral mild-to-moderate sensorineural hearing loss

**Figure 2:**
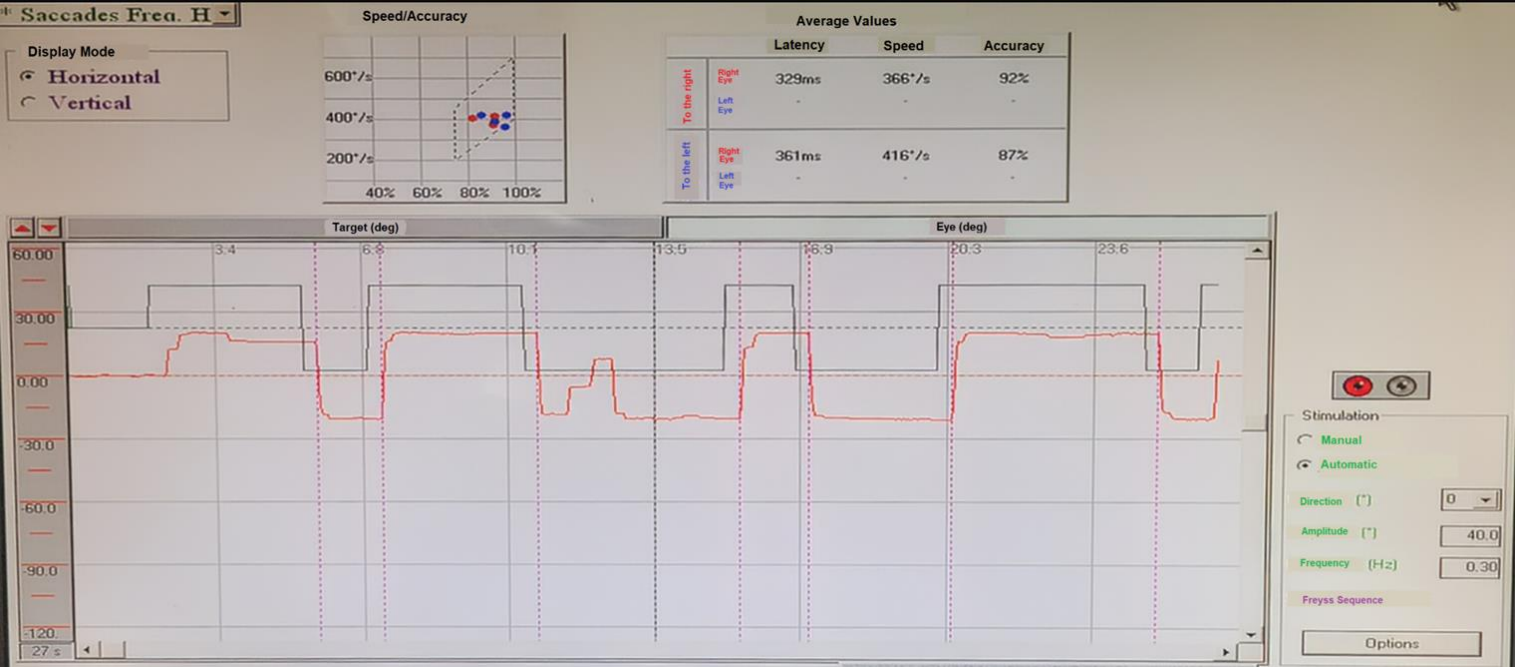
the random saccade test demonstrated a refixation latency exceeding 280 ms in both eyes

**Diagnostic assessment:** tonal audiometry revealed bilateral mild to moderate sensorineural hearing loss, predominantly affecting high frequencies, more pronounced on the right side with an average loss of 40 dB in the right ear and 36 dB in the left ear (Figure 1). Videonystagmography results were as follows:

*Saccade test:* the refixation latency, which separates the appearance of a new target and the onset of the eye's deflection towards it, was greater than 280 ms in both eyes, without interocular dissociation, with normal accuracy (between 70-100 %). Furthermore, the graph shows right hypometria with intra-saccadic ocular phenomena of the right eye (Figure 2).

**Figure 3:**
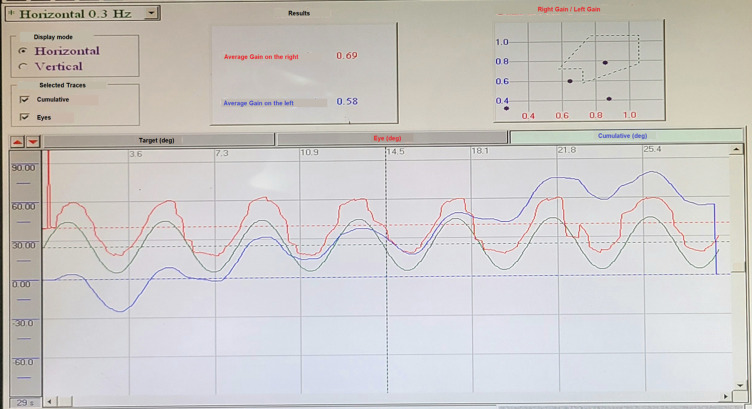
slow eye pursuit test revealing a gain of 0.69 to the right and 0.58 to the left, consistent with roughly symmetrical eye movement

*Slow eye pursuit test:* the gain of the slow eye pursuit test was 0.69 to the right and 0.58 to the left (lower than the threshold of 0.7 in both eyes), but roughly symmetrical. The overall appearance of the curve shows saccadic phenomena, with a predominance of left visual saccades (Figure 3).

**Figure 4:**
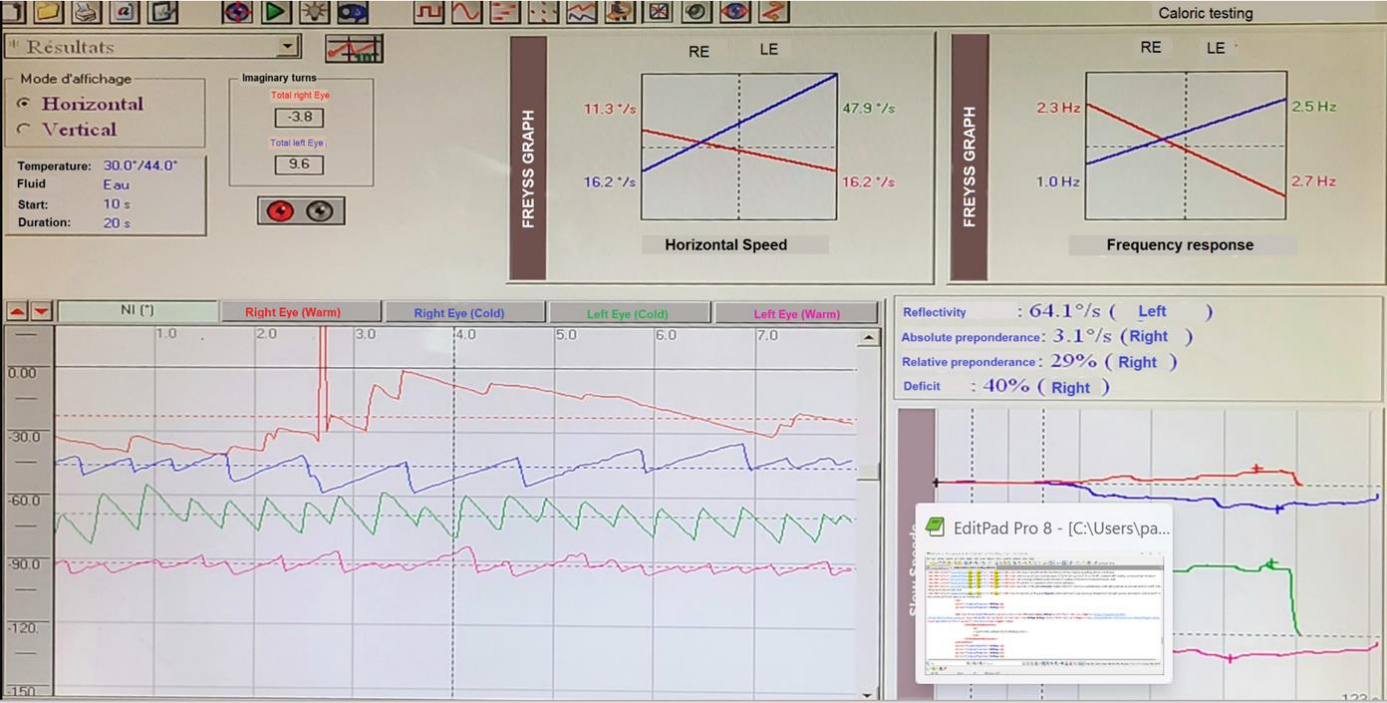
caloric testing revealed a predominance of response in the left horizontal semicircular canal

*Caloric testing:* this test revealed a left canal predominance clearly visualized on the Freyss diagram, with a calculated deficit of 40% on the right and a reflectivity of 64.1°/s on the left, indicating normal reflectivity (Figure 4).

**Figure 5:**
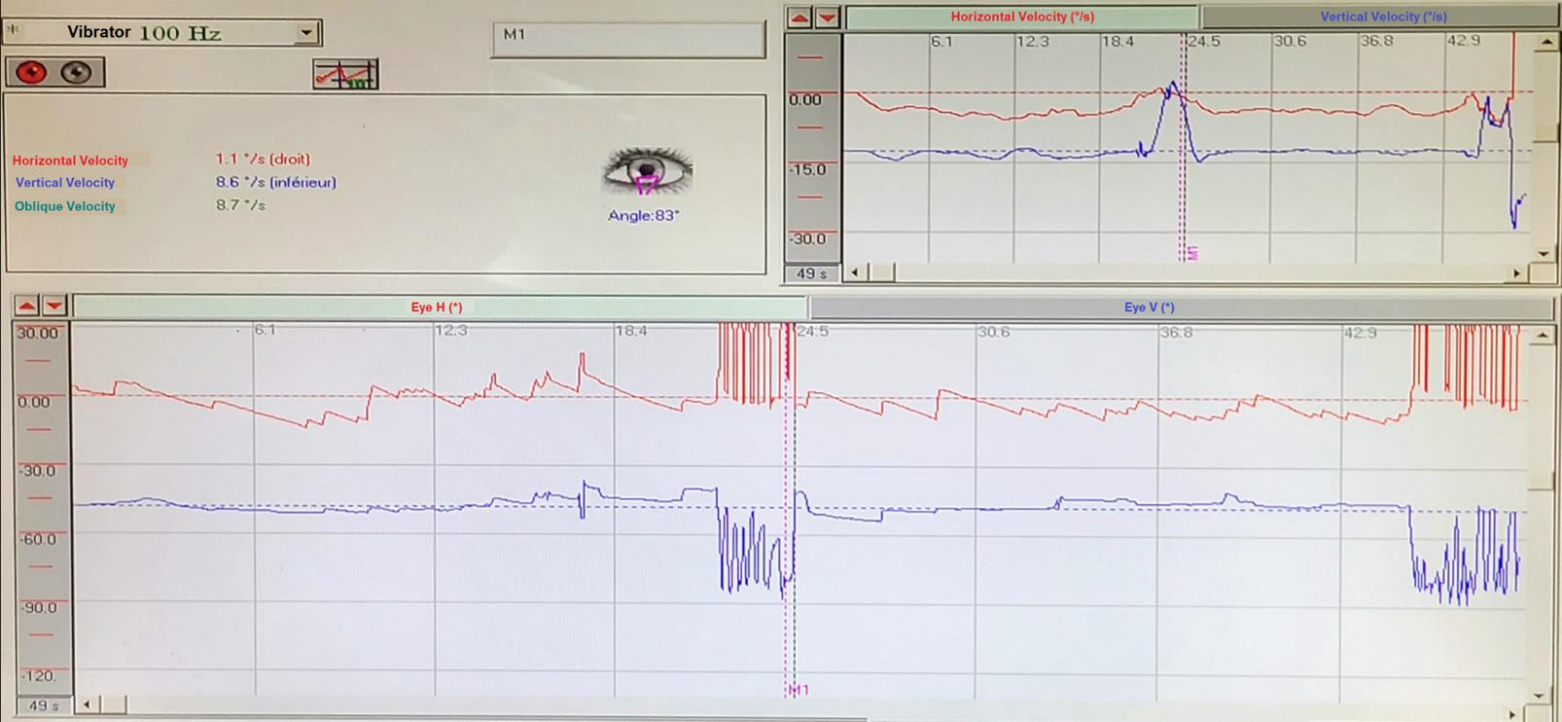
the vibrator test revealed a down-beating nystagmus

**Figure 6:**
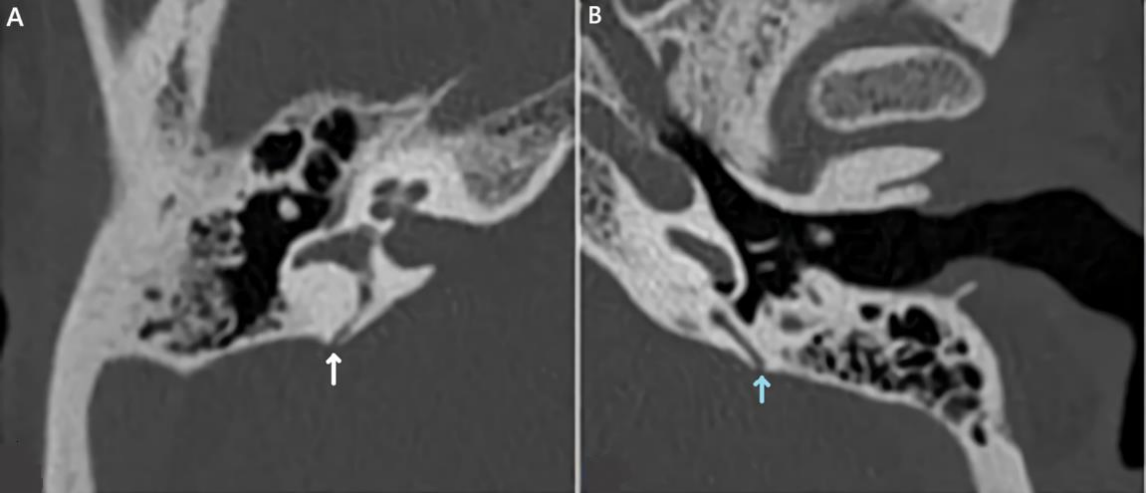
axial view of the patient´s temporal bone CT scan showing dehiscence of the right posterior semicircular canal (A); and (B) of the left posterior semicircular canal

**Figure 7:**
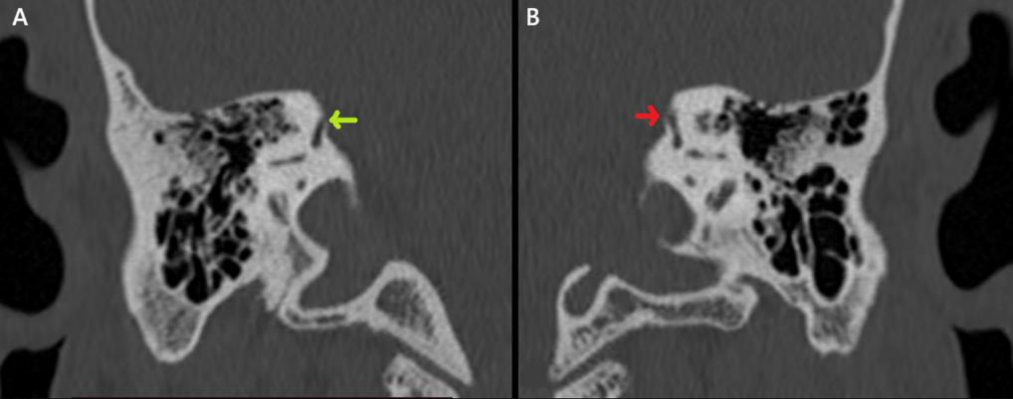
frontal view of the patient´s temporal bone CT scan showing a dehiscence of the right superior semicircular canal (A); and (B) no bone defect of the left superior semicircular canal

*Vibrator test:* this test revealed the presence of a down-beating nystagmus greater than 2°/s induced by the vibrator (Figure 5). All these tests gave us a hint about a possible vestibular disorder. Temporal bone CT scan confirmed the dehiscence of the PSC in both ears and a dehiscence of the SSC in the right ear (Figure 6, Figure 7).

**Therapeutic interventions:** although we found no standard of care recommendations for the management of multiple semicircular canal dehiscences, we presented the classic therapeutic options to the patient, including surgery and the conservative approach. The patient chose conservative treatment. Since hearing loss was a real burden to the patient, we proposed hearing restoration using bilateral behind-the-ear hearing aids attached to a custom ear mould and thin tubing. In this configuration, the speaker is placed inside the external auditory canal and connected to the main device via a thin electrical wire. This arrangement ensures optimal sealing of the external auditory canal, significantly reducing loud noises and consequently the Tullio phenomenon. It also allows for ambient sound processing while maintaining binaural communication between the two hearing aids, ensuring a constant exchange of information between the devices. This enhances the directivity of the microphones, providing improved spatial awareness and sound source localization. Constant communication between the referring physician and the audiologist was crucial in this phase. Three adjustments of the device were performed at one-week intervals to achieve increasingly satisfactory results. We also informed the patient of some lifestyle changes he must make to ensure the best quality of life with his condition, such as avoiding loud noises, using earplugs, and avoiding scuba diving or flying (major pressure changes).

**Follow-up and outcome of interventions:** we followed the patient for over a year, and we observed a significant improvement in the management of his symptoms. The patient reported a marked reduction in vertigo and Tullio phenomenon by strategically turning off the hearing aids when exposed to loud noises, such as while drilling his patients' teeth, while keeping the device in place to effectively seal the external auditory canal. This adjustment helped reduce the Tullio phenomenon, leading to better symptom control, less dizziness and nausea, and an improved quality of life. These findings highlight the effectiveness of non-invasive strategies in managing complex vestibular disorders.

**Patient perspective:** being constantly exposed to loud sounds made my daily work increasingly difficult due to the vertigo and discomfort they triggered, especially given my profession as a dentist. The progressive hearing loss and imbalance I experienced significantly affected both my professional and personal life. Receiving the diagnosis of multiple semicircular canal dehiscence was initially overwhelming, but the collaborative care and tailored conservative treatment, particularly the use of custom-fitted hearing aids, helped me regain control over my symptoms. By adjusting the devices and learning how to manage sound exposure, I´ve been able to reduce vertigo episodes, improve my hearing, and continue working with greater comfort and confidence.

**Informed consent:** an informed consent was taken from the participant before the testing. Consent for publication was obtained from the participant.

## Discussion

Semicircular canal dehiscence is a rare pathology caused by a focal bone defect in one or multiple of the semicircular canals. Minor *et al*. first described this syndrome in the superior SCC in 1998 [1]. The etiology of these anomalies is still poorly understood [2]. Semicircular canal fulfills essential functions for balance and spatial orientation in three-dimensional space. Dehiscence of one of these canals leads to disturbances in the pressure of endo and peri-lymphatic fluids and loss of acoustic energy. When the stapes plate vibrates in response to sound, slight movements of perilymph are generated, transmitted to endolymph, and misinterpreted by the brain as patient movements, causing vertigo symptoms, mainly nystagmus, which is a valuable diagnostic element used in videonystagmography [3]. The SCC oculomotor projections are made to the ipsilateral superior oblique and contralateral inferior rectus muscles for the posterior canals and on the ipsilateral superior rectus and contralateral inferior oblique muscles for the anterior canals [4,5].

Symptoms associated with this condition are variable and nonspecific. However, the most indicative sign is the Tullio phenomenon, described in 1929, where the patient experiences vertigo and/or nystagmus triggered by high-intensity sounds. These symptoms may also occur during pressure changes in the external auditory canal, known as Hennebert's sign [6]. Audiometry often reveals conductive hearing loss. Videonystagmography (VNG) is a valuable tool in diagnosing SCCD by providing objective data on vestibular function and identifying associated abnormalities. It involves several tests: 1) the slow eye pursuit test evaluates eye movement induced by continuously moving visual targets. The ratio between the target's velocity and that of the eye, called “gain,” should not fall below 0.6 [4]. 2) Saccade test evaluates rapid eye movements triggered by the appearance of a visual target in the peripheral retina. Voluntary control is minimal [4]. The precision of the saccade is mainly studied. Therefore, two pathological results can be described: i) hypometria, where multiple saccades are needed for the eye to reach the target, possibly indicating brainstem involvement; ii) hypermetria, where the saccade overshoots its target, indicating cerebellar involvement. In our case, the patient had results suggesting a central origin at the brainstem level, so we further conducted a caloric test and vibrator-induced nystagmus. 3) The caloric test that examines the lateral semicircular canal by irrigating the patient's ear with warm then cold water. The patient is lying down with the head tilted 30° from the horizontal plane, then the patient's ear on each side is irrigated with water at 44°C (warm stimulus) and at 30°C (cold stimulus), which triggers a beating nystagmus on the same side in the warm test and on the contralateral side in the cold test. The frequency of the ocular nystagmus or the amplitude of the slow phase velocity of the ocular nystagmus is then measured and plotted on the Freyss graph [7]. 4) Vibrator-induced nystagmus (VIN): vibratory stimulation at a frequency of 100 Hz applied to the mastoid simultaneously excites the vestibules and both the right and left semicircular canals. In a healthy individual, these bilateral stimulations tend to cancel each other out. There is no central compensation during this test. However, in the presence of vestibular sensory deficit, stimulation of the healthy vestibular sensor induces beating nystagmus towards the healthy side, regardless of the stimulated side. In cases of vertical semicircular canal involvement, down-beating nystagmus is noted for the anterior SCC and up-beating nystagmus for the posterior SCC [8,9]. In our clinical case, we observed the presence of down-beating nystagmus.

Diagnosis of SCCD is confirmed using a temporal bone CT scan that discloses a bone defect, associated inner ear malformations, and study anatomical ear variants with surgical risks. Currently, surgery is the only treatment that addresses SCCD, either by filling the defect to close the canal or by plugging the canal to block it. After these procedures, the patient must undergo vestibular rehabilitation for a few months to adapt. The surgical approach can be either supra-petrous or trans-mastoid. These procedures carry several risks, including vestibular dysfunction, sensorineural hearing loss at high frequencies, labyrinthitis, meningitis, and vestibular ossification. Non-invasive approaches can be used, especially for patients with mild symptoms, where the drawbacks of surgery outweigh the benefits. These approaches include hearing aids, vestibular rehabilitation, and lifestyle changes such as avoiding loud noises and significant pressure changes [10]. Both options must be clearly explained to patients suffering from SCCD, discussing the risks and benefits, including potential side effects.

## Conclusion

In this article, we emphasized the importance of VNG in diagnosing semicircular canal dehiscence (SCD) through a clinical case study of bilateral and multiple SCC dehiscence and a review of the literature. VNG offers an objective and non-invasive approach to evaluating vestibular function and identifying abnormalities associated with SDC. Its systematic use and appropriate interpretation can contribute to early diagnosis and effective management of this rare but debilitating condition. Future studies could further deepen our understanding of the effectiveness of VNG in diagnosing and monitoring multiple and bilateral canal SCD.
